# Exploring Parents’ Violence Against School Teachers: Manifestation, Risk Factors, and Coping Strategies

**DOI:** 10.3390/bs15101429

**Published:** 2025-10-21

**Authors:** Ruth Berkowitz, Naama Bar-On Shmilovitch, Shay Tzafrir, Guy Enosh

**Affiliations:** 1 School of Social Work, University of Haifa, Haifa 3103301, Israel; 2 School of Management, University of Haifa, Haifa 3103301, Israel

**Keywords:** parent violence against teachers, teachers’ victimization, social climate, organizational climate, parent school involvement

## Abstract

Research and public attention on violence directed toward school teachers are increasing. Yet to date, our knowledge on violence against teachers is limited, because most research has focused on student-perpetrated violence, largely overlooking the aggression directed at teachers by parents. To fill this gap in knowledge, this study used a qualitative approach based on in-depth semistructured interviews with 46 teachers, school leaders and policy-level managers to explore the phenomenon of parents’ violence against teachers, including manifestation of the problem, risk factors, and effective coping strategies. Following the principles of grounded theory, the results suggest that parents’ violence toward teachers takes various forms, mostly rudeness, shouting, intimidation, and verbal threats. These behaviors create complex challenges that affect teachers both personally and professionally, requiring coping mechanisms at the societal, school, community and individual levels. Effective strategies encompass improving the social and organizational climate in schools, providing mentoring and support, offering conflict management training for educators, and implementing comprehensive family–school partnership policies at the school level. Implications for research and policy are discussed.

## 1. Introduction

School violence is a continuous and serious public health concern. Decades of research have primarily focused on student victimization, but the past 15 years have seen increasing attention to violence directed toward teachers (e.g., D. Espelage et al., 2013; McMahon et al., 2014; Reddy et al., 2018). Recent findings underscore a troubling global trend of high rates of violence against teachers (e.g., Irwin et al., 2024; Longobardi et al., 2019; Mallory et al., 2024). This issue has significant repercussions, adversely affecting teachers’ health and well-being (Chirico et al., 2021; Olivier et al., 2021; Reddy et al., 2024), exacerbating challenges related to teacher job retention and turnover (e.g., McMahon et al., 2024; Moon et al., 2020; Peist et al., 2024), and consequently posing serious challenges for students and schools. Yet to date, our knowledge on violence against teachers is limited, because most research has focused on student-perpetrated violence, largely overlooking the aggression directed at teachers by parents (Badenes-Ribera et al., 2022). Still, parents interact regularly with schools and teachers, making them vital partners in education. Their engagement can sometimes escalate into aggression due to conflict, high expectations, misunderstandings, and stress (Attanucci, 2004). To address this gap in knowledge, the current research used in-depth semistructured interviews with 46 educators and policy-level managers to explore the phenomenon of parents’ violence against teachers, including manifestation of the problem, risk factors, and effective coping strategies. By understanding the contexts in which parental aggression occurs and how it manifests, identifying common risk factors, and documenting effective coping mechanisms used by teachers and schools, this research aimed to contribute valuable insights to mitigate these incidents. Adopting a constructivist lens (Creswell & Poth, 2015; Flick, 2022), the study emphasized teachers’ subjective interpretations of parental aggression, highlighting how their narratives shape professional identity and everyday practice. The findings can foster the development of targeted prevention programs and interventions that support a safer and more supportive educational environment for teachers, parents, and students alike.

### 1.1. Violence Against Teachers

School violence is defined as any behavior intended to cause harm to individuals or their property, occurring within or outside the classroom, in the vicinity of schools, or on the way to or from school. This broad definition includes face-to-face and electronic media-related verbal and social violence, physical violence, theft and property damage, weapon use, and sexual violence (American Educational Research Association, 2013; UNESCO, 2025).

Although any member of the school community can be a perpetrator or victim of violence, research has predominantly concentrated on peer victimization (Astor & Benbenishty, 2019). Only during the past decade and a half has teachers’ victimization gained recognition as an important and growing public concern. Researchers and practitioners in education recognize that similar to other service professionals such as healthcare providers or social workers, school teachers are also vulnerable to serious violence in the workplace (e.g., McMahon et al., 2024). Teachers may experience various forms of violence in the school setting, with verbal aggression and threatening behavior being the most common and physical or sexual violence and property-related victimization occurring less frequently (e.g., Irwin et al., 2024; Longobardi et al., 2019).

To illustrate, a national survey by the American Psychological Association Task Force on Violence Against Educators and School Personnel involving more than 11,000 U.S. educators and school staff members found that following the removal of COVID-19 restrictions in 2022, between 22% and 80% of respondents reported experiencing verbal or threatening aggression, whereas 2% to 56% reported experiencing physical violence at least once during the year. Students and parents were the most frequent aggressors against teachers, followed by colleagues and administrators (McMahon et al., 2024). However, most research has focused on student perpetrators, leaving a gap in understanding how teachers experience violence from parents. As a result, knowledge of effective ways to support teachers in these situations remains limited.

### 1.2. Factors Contributing to Violence Against Teachers: A Socioecological Perspective on Schools in Context

Building on the socioecological framework of human development (Bronfenbrenner, 1977), educational researchers have suggested that school violence is most accurately conceptualized as the product of dynamic, interconnected contexts that are continually adapting over time. In particular, external factors influencing the school environment interact with internal school dynamics, collectively affecting various facets of school violence and overall safety (Astor & Benbenishty, 2019; D. L. Espelage, 2014). Prior research employing a socioecological lens to explore violence against school teachers highlighted factors associated with teachers’ victimization at the individual, school, community, and societal levels (McMahon et al., 2017).

At the individual level, research has suggested that younger teachers and those with less experience in the profession have greater risk of teacher-directed violence (Martinez et al., 2016; McMahon et al., 2014). Teachers’ classroom management abilities have also been highlighted in the literature as critical for preventing teachers’ victimization. Evidence indicates that effective classroom management and the establishment of clear boundaries by teachers reduce students’ misbehavior and aggressive interactions with educators (Gage et al., 2016) while also preventing conflict and mitigating the risk of violence from students (McMahon et al., 2020). Less experienced teachers often struggle to articulate their classroom management techniques effectively, because these skills typically require time and practical experience to cultivate; furthermore, students tend to exhibit diminished respect toward teachers who appear less confident and lacking in experience (Berkowitz et al., 2022).

A prominent school-level factor consistently associated with reduced violence and greater safety is positive school climate (Thapa et al., 2013). Positive school climate is broadly defined as the “quality and character of school life, which includes norms, values, and expectations that support people feeling socially, emotionally, and physically safe” (Cohen et al., 2009, p. 182). It has been associated with lower rates of violence directed toward teachers (D. Espelage et al., 2013; Martinez et al., 2016). Interpersonal relationships are fundamental components of the school climate. In particular, positive parent–teacher relationships are central to teachers’ victimization. A deterioration in teacher–parent relationships was linked to negative outcomes reported by teachers, including mistrust and a dysfunctional relationship, which can catalyze parental aggression against teachers (McMahon et al., 2023).

Meaningful parental school involvement has also been highlighted in the literature as a fundamental school climate aspect. Epstein (2019) identified six types of parental school involvement and partnership, including helping families create supportive home environments, fostering two-way communication about student progress, organizing parent volunteers, providing resources for learning at home, involving parents in decision-making and leadership, and collaborating with the community to strengthen school programs and support student success. These interconnected approaches work together to build strong partnerships among families, schools, and communities (Epstein, 2019). Active parental involvement, coupled with strong, positive relationships with teachers, plays a critical role in enhancing children’s academic achievement, fostering healthy development, and promoting overall educational success (Boonk et al., 2018; Sheldon, 2019). Meaningful parental involvement has also been associated with a more positive school climate (Berkowitz et al., 2021) and a reduction in school violence (Lesneskie & Block, 2017).

Nonetheless, parental excessive involvement and interference in educational practices may threaten teachers’ authority, undermine their professional autonomy, disrupt classroom dynamics, hinder student independence, and sometimes manifest as disrespect or aggression toward teachers and the educational system (Addi-Raccah & Grinshtain, 2018; Lasater, 2016). Disputes over disciplinary practices are a serious source of tension between parents and teachers that may prompt some parents to act aggressively toward teachers (Badenes-Ribera et al., 2022; McMahon et al., 2023). Limited research has indicated that parental overinvolvement and interference in instructional efforts may increase the risk of parental violence against teachers (Addi-Raccah & Grinshtain, 2018; Tiesman et al., 2014).

An essential element of a healthy school climate is the implementation of explicit, equitable policies concerning violence, accompanied by uniform enforcement. Schools that cultivate a positive climate typically emphasize safety and structural order, creating an environment in which disciplinary measures are applied assertively yet consistently and justly (Berg & Cornell, 2016). Parental violence toward teachers has been associated with a lack of support for teachers’ authority, unstructured regulations, and inconsistent enforcement of school rules (McMahon et al., 2023).

The school’s organizational climate is especially relevant to teachers’ victimization. Previous research indicated that a lack of collegial, managerial, and administrative support and backing, along with negative workplace relationships, significantly contributes to increased violence directed at teachers (Berkowitz et al., 2022; McMahon et al., 2020).

At the community level, parents are vital stakeholders. Adverse parent–child relationships, characterized by recurrent conflicts and poor communication, have been shown to predict dysfunctional relationships with teachers (Leone et al., 2000). Diminished parental authority over children has been associated with increased teacher-directed violence (Berkowitz et al., 2022; McMahon et al., 2020). The socioeconomic status of the community has also been explored in relation to parental violence against teachers. Some evidence suggests that in more affluent communities, parental involvement is often more pronounced, sometimes accompanied by conflicts characterized by threats, verbal aggression, and dismissive or critical attitudes toward teachers that undermine teachers’ professional authority (Addi-Raccah & Grinshtain, 2018; Horowitz, 2009). For example, McMahon et al. (2023) documented instances in which parental aggression towards teacher, fueled by disagreements over their child’s academic decisions, included verbal hostility and threats of legal action.

Finally, society-level factors have also been associated with violence against teachers. A lack of respect and low societal value assigned to the teaching profession can foster negative perceptions, hostility, and violence directed at teachers (McMahon et al., 2020).

### 1.3. Strategies for Addressing and Coping with Violence Against Teachers

A significant gap in research persists regarding interventions designed to address and reduce violence against teachers. To address school violence not specifically related to incidents against teachers, educators and schools may implement various strategies, including exclusionary disciplinary measures such as suspending involved students (Skiba et al., 2022). Although schools frequently use suspensions in response to violence, research evidence suggests that such exclusionary practices can be harmful (Skiba et al., 2014). Security measures, including the deployment of police officers, school resource officers, security guards, metal detectors, and electronic monitoring systems, are also widely implemented, particularly in the United States. Nonetheless, students and educators have expressed that such measures are relatively ineffective in preventing school violence (Astor et al., 1999). In contrast, approaches that incorporate proactive relational strategies, such as prevention initiatives and school climate improvements, have demonstrated positive effects in reducing school violence globally (Bradshaw et al., 2021). To illustrate, a whole-school approach encompassing strong leadership, clear laws, adequate resource allocation, the creation of safe and inclusive environments with well-defined policies and accountability, and the development of partnerships with families, communities, teachers, and students has been shown effective in reducing school violence (UNESCO, 2019). Previous research exploring teachers’ perspectives indicated that educators consider such prevention practices to be the most effective in reducing violence against teachers (Perry et al., 2023).

Given that certain school safety practices have been demonstrated to be ineffective in mitigating overall violence, particularly violence against teachers, there is an urgent need for research to document safety measures implemented by schools and educators. Such research is essential to inform and guide the development of more effective, evidence-based school intervention strategies aimed at reducing parental violence against teachers.

In summary, the present study aimed to explore how parental violence against school teachers manifests, the risk factors associated with this phenomenon, and the common strategies used to address it, as perceived and understood by educators and policy-level decision-makers.

## 2. Method

### 2.1. Study Design and Procedure

This study drew from broader mixed-methods research examining the incidence, extent, underlying causes, and outcomes of violence against school teachers in Israel. Specifically, the data analyzed in this study came from the initial phase of the broader project, during which we used qualitative methods to explore the perceptions and interpretations of school violence—particularly violence against teachers—among teachers, school principals, and policy-level managers, such as directors of educational psychological services and representatives of teachers unions. Given the paucity of research on parents’ violence toward teachers, a qualitative approach was chosen to provide an in-depth exploration of professionals’ insights surrounding this problem. This approach enabled participants to express their observations and insights with depth and contextual detail.

Grounded in qualitative methodology, the study adopted a constructivist approach (Flick, 2022), which emphasizes understanding experiences through the meanings individuals assign to them. This lens is particularly valuable for examining parental violence against teachers, because it enables a close analysis of how teachers interpret and respond to such events and how these interpretations shape their professional identity and practice (Creswell & Poth, 2015). Teachers’ narratives, influenced by their training, experience, and personal beliefs about education and parent–teacher relationships, can reveal how they assess risks, perceive threats, and develop coping strategies, providing insight into the personal and professional adjustments prompted by these encounters (Denzin & Lincoln, 2011).

### 2.2. Sampling and Participants

Theoretical sampling was used to achieve the most varied sample possible of school principals and subject and homeroom teachers working in postprimary public schools from both the Hebrew and Arabic language sectors in the official Israeli school system. Participants were identified using informal networking and the assistance of professionals working in schools across Israel. We first reached out to school principals and presented the project. Principals who agreed to participate in the research introduced it to their teachers. All teachers and school principals who agreed to participate and were available at the appointed time were recruited for the study. Additionally, purposeful sampling was employed to include individuals in policy-level managerial positions and an academic scholar. We further engaged policy-level managers from educational psychological services and a teachers union, inviting them to participate in the study.

The sample featured 46 individuals, including 36 teachers and four school principals from 11 public postsecondary schools in Israel, representing both Hebrew and Arabic language sectors. Additionally, the sample included three managers from educational psychological services and the chair and two coordinators from the teachers union. Most participants were women (*n* = 36, 78.2%), with average professional experience of 14.11 years (*SD* = 9.19). Among those who reported their educational attainment, more than half (*n* = 37, 56.7%) held a master’s degree, approximately one fifth (*n* = 8, 21.6%) held a bachelor’s degree, about one tenth (*n* = 4, 10.8%) possessed a PhD, and two participants (5.2%) were certified teachers. For a comprehensive demographic overview, refer to Table 1.

### 2.3. Process and Data Analysis

This research employed in-depth semistructured interviews to explore participants’ personal experiences and perceptions concerning the studied phenomenon. The interview protocol was carefully crafted by the research team through an iterative process, involving collaboration with experts in school violence, educators, and policymakers. The project’s principal investigators conducted the interviews. Interviews started with a broad, open-ended question, encouraging participants to share their views, understanding, and firsthand experiences related to violence against teachers. When needed, this initial question was followed by targeted inquiries regarding risk factors, antecedents of violence against teachers, outcomes of incidents, coping strategies, school policies, and responses. Most interviews ranged from 1 to 2 h in length. All sessions were audio recorded and transcribed verbatim to enable detailed cross-sectional analysis.

Data analysis was conducted following the principles of grounded theory, as outlined by Strauss and Corbin (1998), which emphasize researcher engagement and active participation in the analysis process, guided by a preliminary understanding of the research area. The analysis proceeded through four distinct phases. Initially, two researchers carefully reviewed the transcripts multiple times to develop a comprehensive understanding of the dataset, allowing preliminary insights to emerge. In the second phase, meaningful segments of the text, or units of meaning, were identified to align with the study’s objectives. The third stage involved grouping similar units of meaning through axial coding, which revealed relationships between categories and subcategories related to both context and content (Strauss & Corbin, 1998). The analysis was conducted using ATLAS.ti software (version 8.4.25), which facilitated the extraction of meaningful segments and organized the data into thematic categories (Friese, 2019). Finally, thematic connections were made to synthesize a holistic representation of the data, culminating in the development of typologies and substantive insights rooted in participants’ narratives (Creswell & Poth, 2015).

### 2.4. Trustworthiness and Credibility

To uphold the principles of trustworthiness and methodological integrity, the study conformed to the qualitative research standards established by Lincoln and Guba (1985) and Shenton (2004). Credibility was secured through comprehensive data triangulation, incorporating diverse perspectives from educators, professionals operating in schools, and policy-level managers. The analytic process involved three researchers who independently coded the data and then compared and reconciled their interpretations, providing an internal validation mechanism that strengthened the reliability of the thematic framework. Transferability was strengthened by providing detailed descriptions of participant demographics, recruitment strategies, and interview procedures. Dependability was ensured via continuous discussions in the research team, which facilitated critical reflection on potential biases and maintained consistency in coding practices across the dataset. Finally, confirmability was attained by thoroughly documenting all analytic decisions, engaging in peer debriefing, and ensuring that themes were directly derived from the data, minimizing influence from preexisting researcher assumptions.

At the beginning of each interview, participants were informed that their participation was voluntary, and they were free to discuss or refrain from discussing any issues they wished. They could also withdraw from the interview at any point. All personal identifying information was kept confidential; results are presented using pseudonyms.

## 3. Results

Following the principles of grounded theory, the analysis of participant interviews suggested that teachers engaged with various stakeholders in the school community, including colleagues, students, and students’ parents. Although typically viewed as external stakeholders, parents were also capable of perpetrating violence against educators. The results suggest that this aggression took various forms, from rudeness to shouting, intimidation, and verbal threats. Such behavior presented complex challenges that affected teachers both personally and professionally, necessitating coping mechanisms at both institutional and individual levels. The following themes explore how parents’ violence toward teachers manifested, associated factors, and the strategies teachers employed to manage such encounters.

### 3.1. Many Faces of Parental Violence Against Teachers

The findings indicate that verbal victimization by parents was the most common form of aggression experienced by teachers. Teachers frequently described encounters characterized by rude, contemptuous, and hostile communication. These incidents frequently stemmed from disciplinary actions directed at students, which escalated into confrontations with parents. For instance, Amira (English teacher) recalled a confrontation marked by aggression that occurred following the removal of a disruptive student from her class:

“Last year marked the first time I encountered verbal and almost physical aggression from a parent. The mother is well-known for her aggressive behavior. After I asked her son to leave the classroom for misbehavior, she immediately called her mother. She then came to the school, shouting and cursing at me”.

Teachers also reported experiencing harassment and threats, often intended to undermine their authority or pressure them into altering professional decisions, especially concerning student misbehavior and disciplinary issues. Sharon (special education teacher) shared:

At my previous school, I received terrifying threats, parents yelling over the phone: “If you don’t let my son in, I don’t know what I’ll do to you! I’ll bring whoever I need!” One father, with a known history of aggression, shouted, “Don’t talk to my son like that! I’ll mess you up.”

These threats frequently extended beyond the school context, affecting teachers’ personal lives. Ravit (math teacher) recalled being pursued beyond school grounds by a mother whose son had been suspended for fighting, highlighting the emotional toll and heightened sense of insecurity that such confrontations can impose on educators. “One mother stalked me, kept calling, threatened to get me fired, said she’d take revenge. She waited for me at the school gate. I felt incredibly unsafe.”

The analysis revealed that one of the most prevalent threats these teachers encountered involved parents’ bypassing their authority, often threatening to escalate issues to the school principal when dissatisfied, thereby undermining trust between teachers and families. For example, Sarit (teacher) recalled a mother who reacted this way after being denied a request for extended exam time for her son: “One mother insisted that her son needed extra time on an exam, even though there were other reasons for his declining performance. When I didn’t agree, she said, ‘If you won’t handle this, I’ll go straight to the principal.’”

Parental threats frequently involved external entities such as legal representatives, representatives from the Ministry of Education, district officials, or even the media when dissatisfied. Per study participants, they employed these tactics to intimidate teachers, interfere, and influence their professional decisions. Noga (class coordinator) described instances in which parents sent legal warning letters as a means of exerting pressure; these threatening letters significantly undermined teachers’ professional authority: “Sometimes we receive letters from lawyers meant to intimidate us. It’s their way of pressuring us to change our decisions. … It’s paralyzing; you can’t educate children when you’re being legally harassed.”

Bypassing teachers’ authority eroded trust and strained the relationships between teachers and parents. Dana (pedagogical coordinator from the teachers’ union) shared her perspective on this concerning trend:

“A significant flaw in our system is the tendency of some parents to bypass direct communication with teachers or school staff and instead escalate their concerns directly to the Ministry of Education. The complaint then travels from the minister to the director-general, to the district superintendent, to the school supervisor, and ultimately to the principal, who often remains unaware of the issue due to a lack of initial direct communication with the school.”.

These findings highlight the dual challenges that these teachers faced: Parental threats toward teachers directly undermined their professional functioning, whereas fear of retaliation further discouraged them from addressing students’ misconduct. Shirin (special education teacher) noted: “I’m afraid to call parents and tell them what their child did. My students misbehave, but I hesitate to report it to their parents.”

Not only did teachers face verbal aggression and threats from parents, but they also encountered intimidating disruptive behavior. Natalie (school principal) shared instances in which parents entered classrooms and interrupted lessons without restraint in front of the class.

“In the past 6 years, there’s been a noticeable rise in parents’ audacity. We’ve had parents barging into classrooms. There is a noticeable decline in restraint, as parents now often interrupt lessons directly to speak with teachers rather than waiting for an appropriate time”.

The findings show that teacher-directed aggressiveness also manifested through digital spaces, particularly via WhatsApp. Ravit (math teacher) observed that parents frequently send messages during inappropriate hours, expecting an instant response:

“There’s a lot of harassment on WhatsApp. Parents feel free to message you whenever it suits them, 6 in the morning, 6:20, 11 at night. There are no boundaries at all. They expect immediate answers and don’t care that the timing is completely inappropriate”.

Several interviewees observed that conflicts with parents tend to escalate more intensely than those with students, largely because of parents’ heightened emotional investment and overprotectiveness. Rina (director of counseling psychological services) explained that parental anger often escalates at school, driven by distress or a sense of injustice:

“It also affects people on a very personal level. Parents have expectations about how their children should be treated. … We see intense parental anger at school, often sparked by a perceived injustice toward their child. It’s usually directed at teachers and principals and stems from distress, insult, or a sense of unfairness.”.

### 3.2. Factors Associated with Parental Violence Against Teachers

Regarding aggression faced by teachers, the analysis demonstrated that these incidents did not occur in isolation from factors both inside and outside the school. The findings indicate multiple risk and protective factors across individual, school, community, and societal levels that facilitated the emergence and escalation of conflicts and violence from parents toward teachers. These factors influenced the interactions among teachers, parents, and students, potentially eroding teachers’ professional authority and compromising their sense of safety at school.

### 3.3. Individual Factors

**Teachers’ Ability to Establish Boundaries with Parents.** Ella (history teacher) reflected on her ability to set boundaries and maintain composure in challenging interactions with parents. She said this is an important individual characteristic that helps her navigate difficult situations: “I have no problem speaking with parents. I’m responsible for every word I say. But if a parent yells, I say, ‘Goodbye, call back when you’re calm,’ and I hang up. I never raise my voice.”

**Number of Years in the Profession.** The findings suggest that more experience and seniority can enhance teachers’ capacity to set firm boundaries with parents, especially in situations involving aggression or escalating conflicts. This capacity depended not only on individual personality traits but also on confidence and practical knowledge accumulated through years of experience. Sigalit (deputy principal) recounted an incident in which she successfully intervened to deescalate a conversation, noting that younger teachers often find these situations challenging due to their limited experience:

Just before you came in, I spoke with a student’s mother, and the conversation got intense. I told her, “I’m stopping this conversation. You can’t speak to me like that.” It’s not easy to do that, especially for young teachers. Today, parents feel entitled to speak aggressively and disrespectfully, but they are more reluctant to do so with experienced teachers.

Ravit (math teacher) similarly reflected on the challenges she faced during the early years of her career, describing them as the most difficult period of her teaching journey. She highlighted how her lack of knowledge and experience often compelled her to confront challenging situations in front of students and parents, sometimes leading to inadequate responses due to her inexperience:

The first three years when I started teaching were the hardest years of my life in terms of teaching. It was really like jumping into the deepest waters. When I started, I was given the role without knowing the system, without knowing the school, difficult work environment. I encountered many challenging situations with parents and often handled things incorrectly because of a lack of knowledge and experience. It was one of the hardest periods I’ve had, but it also gave me many tools for coping better with parents and students, assertiveness, and other skills.

### 3.4. School Factors

#### 3.4.1. School Social Climate

**Teacher–Parent Relationships.** The degree of trust between parents and teachers emerged as a crucial element of the school’s social climate. Schools in which stakeholders fostered this trust experienced stronger bonds, greater mutual respect, and a reduction in potential conflicts. Samaa (Arabic teacher) explained:

In our school, there is a personal approach. Every teacher, every teacher who is assigned a class is required to conduct home visits, so the teacher gets to know the parents, the home, and the student’s environment. … This is something that greatly strengthens and brings in the teacher, the parent, and the student.

This illustrates how proximity and familiarity served as preventive mechanisms against hostility. Conversely, when parents lacked confidence in the education system, their attitudes were more adversarial. Keren (English teacher) reflected on how parents’ perceptions shape their behavior: “I think it starts with the image that parents have of us. If parents don’t trust the system, don’t think it is good for the children, and see it as a factory, then there may be more aggression.”

The consequences of deteriorated teacher–parent relationships became especially clear when parents were aggressive and unwilling to collaborate. Oshrat (English teacher) described one such situation:

I have a student who has really deteriorated academically; he does nothing in class. Now, this creates a situation where the parent is aggressive toward you, and there is a certain tension and unwillingness to cooperate on the parent’s part. The process does not advance the child. You cannot do it without the parents. In such a case, it is very difficult, almost impossible, to advance the student.

**Parental School Overinvolvement.** A dominant theme across interviews was the centrality of excessive parental school involvement and interference in teachers’ professional decisions as an antecedent of teacher-directed violence by parents. Grades and academic performance often served as triggers for parental aggression. Teachers consistently described situations in which grades became a site of negotiation, pressure, and even threats. Amal (Hebrew teacher) recounted an incident highlighting parental reactions to grades: “There was once a mother who started sending me a very rude message with verbal abuse on WhatsApp because her child didn’t get a good grade.” Similarly, Shirin (special education teacher) described a more severe scenario that illustrates the emotional impact of parental pressure on teachers:

I had a student whom I gave a 95 on a test. He threatened me, he shouted. I was trembling, and I almost cried. I left the classroom, and he followed me with his phone, with his father shouting, “Dad wants to speak with you.”

These examples suggest that the academic domain is not only a source of tension but also a symbolic battleground where parental authority clashes with the teacher’s professional judgment. Pressure to raise grades, sometimes accompanied by intimidation, illustrates how academic evaluation becomes a flashpoint that destabilizes teacher–parent relationship and trigger aggression toward teachers.

**Disciplinary Rules, Consistent Procedures, and Enforcement.** The findings indicate that a clear and consistent school policy regarding violence functioned as a protective factor for teachers, shaping parents’ expectations and reinforcing the legitimacy of teachers’ authority. Analysis of the interviews suggests that the existence or absence of such procedures directly influenced the quality of parent–teacher relations and risk of aggression. In schools that defined clear boundaries and explicitly communicated the message that violence is unacceptable, teachers reported greater safety along a strong sense of institutional support and security. Hagit (teacher) highlighted the importance of clear messaging regarding violence:

In our school, it is very clear that violence is a red line. Every year at the beginning of the school year, parents receive information about the school, and among other things, this issue of violence is addressed. … For us, it is a red line, and we treat it with the utmost severity. Parents know that we do not overlook any incident of violence that occurs here.

By contrast, in schools in which each teacher acted according to personal discretion without a consistent policy, an atmosphere of ambiguity and inconsistency emerged, creating fertile ground for recurrent aggression. Amal (Hebrew teacher) reflected on the consequences of inconsistent disciplinary practices:

I’ll tell you what the serious problem is: In our school, there is no uniform policy for all teachers. Each one wants to gain the parents’ approval, and we don’t follow a consistent policy. … I often say that the violence we see stems from the fact that we are not handling incidents properly.

These accounts suggest that a lack of uniformity not only weakens enforcement but also conveys to parents that the system is negotiable, thus legitimizing confrontational behavior.

Teachers highlighted that disciplinary measures are not merely technical procedures but also symbolic acts that communicate the school’s priorities. In some schools, consistent disciplinary enforcement created a collective sense of responsibility and protection. Sigalit (deputy principal) recounted an example of collaborative intervention:

We work very closely, what you might call a tight network. We don’t deal with it alone; we consult with one another, and of course, we invite the parents and keep them informed. We don’t just let it slide. There’s no such thing as an incident happening and us skipping over it or giving it up.

Here, participants framed discipline as collaborative, engaging both teachers and parents in a structured process. In contrast, other teachers reported that attempts to please parents led to diluted responses, weakening the perceived seriousness of violent incidents. Shirin (special education teacher) commented on the tendency to compromise institutional standards to avoid parental conflict: “The general policy [in my school is], how would you describe it? Is it to blur issues? Sweep problems under the rug? It is done to appease both the children and their parents.” This tendency to prioritize parental satisfaction over consistent enforcement reflected a broader erosion of institutional authority, whereby the avoidance of conflict undermined teachers’ sense of security.

#### 3.4.2. School Organizational Climate

**Insufficient Systemic Support and Lack of Effective Tools.** A recurring risk factor noted by interviewees is the sense of helplessness that teachers experienced when confronting parental aggression. Unlike conflicts with students, for which established disciplinary measures provide guidance, teachers lacked effective tools to manage encounters with parents. Shay (biology teacher) reflected on this challenge:

At my previous school, a student pushed a desk at me. When the mother was called in, she began yelling. While the principal supported me, he admitted, “There’s really nothing we can do.” When a parent yells at you, you can’t remove them from the school or send them out of the classroom. There are no effective options available to address such behavior.

Sarit (teacher) observed that because of the lack of effective tools to manage parental aggression, teachers often resort to avoiding direct confrontations with aggressive parents as a coping strategy. “Conversations with parents can turn unpleasant, and there’s often aggression with no consequences. Teachers just try to avoid dealing with such parents again. You can’t call a parent in for discipline.”

**Insufficient Collegial Support.** An additional critical risk factor for parental violence against teachers is a negative organizational climate, characterized by a lack of systemic support and inadequate backing for educators dealing with parental aggression. Interviewees noted limited support from school leaders and the Ministry of Education, with a tendency to prioritize maintaining “industrial silence” over addressing issues. Oshrat (English teacher) described school management as frequently hesitant and fearful of parental repercussions, often unwilling to intervene: “There are many situations where you feel unsupported. When I see news about teachers being slapped or punched by parents, I get it. Sometimes, the school leadership is simply afraid of parents. It happens often.”

Yulia (English teacher) provided a telling example: After a student was not promoted to an honors group due to academic criteria, the parents appealed to the principal, who ultimately overruled the teacher’s decision.

After meeting with me, the parents went straight to the principal. Five minutes later, I was called in and told, in front of them, “Move her to the honors group.” It was bullying. I was furious. That incident is one of the reasons I stepped down as coordinator.

Negative organizational climate extends beyond the school environment, encompassing the policy level. Interviewees criticized the Ministry of Education for its inadequate support, contributing to the failure to protect teachers adequately. Nadav (school principal) expressed his frustration: “If parents exert enough pressure, the Ministry of Education often sides with them, even in cases of severe violence, because they fear parents and media exposure. That leaves teachers unprotected.”

To uphold a culture of industrial silence, Dana (pedagogical coordinator from the teachers’ union) observed that the Ministry of Education frequently ignores teachers’ professional opinions and offers insufficient support, ultimately undermining their status and authority in the eyes of students and parents.

If a student wants to study advanced math, even if you think they’re not suited for it, the ministry says, “Do what the student wants.” Where is the teacher’s voice? What about teachers’ professional experience? If the ministry disrespects teachers, why wouldn’t parents? Everyone’s just trying to avoid conflict.

### 3.5. Community Factors

**Overprotective Parenting.** Interviewees largely attributed the increase in school aggression to a shift toward protective parenting, characterized by overreach, persistent interference, and attempts to influence internal school decisions. Ella (history teacher), recalled a parent’s aggressive demand for accommodations following a didactic diagnosis, exemplifying how protective parenting frequently encroaches on school decision-making processes:

In recent years, parental intervention has increased significantly. For example, when a child is diagnosed [with learning or attention deficit disorders], parents immediately demand all recommended accommodations. This often leads to arguments and shouting. Parents today are highly aggressive, believe they and their children are entitled to everything, and rarely let their children cope independently—what we call helicopter parenting.

Tamar (director at psychological counseling services) described this parenting style as one in which parents prevent their children from independently navigating the education system, instead stepping in and taking control of their interactions with the school.

Today, parents are very protective of their children. They give them fewer chances to deal with things independently and intervene more. … Teachers are threatened by parents who say they’ll bring a lawyer. There is much more intimidation today. … Parents, no less than children, are opinionated toward schools, less respectful.

**Declining Parental Authority.** Although interviewees noted that some parents overprotect their children and interfere with teachers’ educational efforts, they also highlighted a decline in parental authority over children as a significant risk factor.

This trend is characterized by blurred boundaries, diminished parental control, and an attempt to shift educational responsibilities to the school. The weakening of parental limit-setting increases the school’s burden and can contribute to more frequent conflicts with parents. Hagit (teacher) described this dynamic:

Over the years, I see that parents are losing their authority over their children. … Parents tell me, “You tell them,” as if passing the responsibility. I say, “With all due respect, I’m only the teacher. I didn’t give birth to the child. I’m not their mother or father.” You see that the parent cannot set firm boundaries for the child.

**Community Socioeconomic Status.** Interviewees further emphasized the role of the community’s socioeconomic characteristics as a contributing factor in aggressive parental behavior. Schools located in affluent areas, for example, often face highly assertive or confrontational parents who have the means to hire legal representation and make formal demands. Anat (Arabic teacher) described parental aggression in schools located in affluent areas:

If you ask me about schools in other areas, especially those located in very affluent communities, you see a lot of parental aggression toward teachers. … They hire lawyers, send threatening letters to teachers, and say, “Now you educate the children like this,” effectively tying our hands. It’s a serious problem.

**Family Disruption and Crisis.** Participants described familial adversity and crisis, including parental divorce and a history of criminal involvement, as increasing the likelihood of conflict with parents. Ravit (math teacher) reflected on how family dysfunction, crisis, and stress can increase parents’ tension and aggression toward teachers:

One case was last year, I had a student from a difficult family background. The parents were divorced; the father had a criminal past and had been in rehabilitation. The mother is raising all three children alone, with a very challenging family background. The child lacks motivation to study, swears, acts disrespectfully, and doesn’t let the teacher teach. When trying to reach out to the mother in an attempt to engage her for the sake of her child, she cursed me, saying that I’m worthless, a bad teacher, blamed me for her son’s problems, and yelled at me that she is moving her son away from me, saying that I have no idea how to deal with children.

This account exemplifies how familial instability and dysfunction can contribute to behavioral issues among children and subsequently increase tensions and aggressive responses toward teachers. Such backgrounds may foster feelings of frustration, helplessness, or anger among both parents and children, which can manifest as conflicts with teachers.

### 3.6. Societal Factors

**Diminished Appraisal of the Teaching Profession.** Interviewees highlighted a decline in the social perception of teaching, which can reduce respect for teachers and foster aggression. Sarit (teacher) explained: “Everyone has been to school and knows teachers. There’s a lack of respect for teachers. This stems from society’s view that teaching isn’t a big deal. It’s seen as a job for those who didn’t succeed elsewhere.” Oshrat (English teacher) described how parents convey this low regard to their children:

Parents today belittle teachers a lot. You hear parents tell their kids, “Anyone can be a teacher these days; it’s not special like before.” This message is passed on to children, teaching them that teaching is not a respected profession.

Several interviewees noted a historical decline in teachers’ authority, leaving them vulnerable to disrespect. Parents sometimes side with their children against teachers. Arie (teachers union) illustrated this point:

My parents saw teachers as almost godlike. The teacher was always right, no matter what. Today’s parents are educated and know the system. They dare to say, “What does this teacher know? I know my child, and they’re right.” They fully support their child. Then the parents tell the child, “Don’t worry, I’ll solve your problem.”

### 3.7. Effective Coping Strategies

Considering the identified risk factors, these teachers often found themselves vulnerable and insufficiently protected in the face of parental aggression. In response to these challenges, the interviewees developed strategies aimed at helping them navigate and cope with such confrontations more effectively.

#### 3.7.1. Establishing Clear Boundaries

The interviews revealed that teachers who effectively managed parental aggression often assertively set and maintain clear boundaries. This involved an immediate response to inappropriate behavior, particularly verbal aggression. Sigalit (deputy principal) recounted a recent incident with a parent whose tone became aggressive during a phone call. She responded by firmly ending the conversation:

A few minutes ago, I spoke with a student’s mother, and the conversation escalated. There was no aggression, but the tone was unacceptable. I told her, “I’m ending the conversation.” Once I said that, her tone changed. But not everyone is capable of doing that.

Interviewees also underscored the principal’s crucial role in setting boundaries with parents. Ilana (school counselor) described how her principal confidently reasserts the school’s authority: “Our principal is very experienced. He knows how to reset them. He says, ‘You chose to enroll your child here, trust us to do what’s best.’”

Nadav (school principal) outlined his clear policy against verbal aggression, demonstrating how such behavior is addressed swiftly and firmly:

In my four years as principal, there have been few incidents where teachers felt threatened. Early on, some parents yelled at a teacher, and I made it clear: That’s unacceptable. I told them they could find another school. The district supervisor came and facilitated mediation but ultimately backed us up: We don’t accept that kind of language.

Dorit (school counselor) highlighted the importance of signaling to parents that teacher safety is nonnegotiable: “Protecting teachers means drawing a line. We [tell parents that we] educate your children, but we won’t allow harm to a teacher. That’s a powerful message from the school.”

#### 3.7.2. Establishing a Positive Organizational Climate

A central factor in preventing aggression and fostering teacher resilience is strong organizational backing. When school leaders and staff members stood firmly behind teachers, it reinforced clear boundaries against aggressive behavior. Sigalit (deputy principal) underscored the school’s commitment to addressing aggression promptly and thoroughly:

Any teacher experiencing aggression receives full support from the educators, the school principal, and the leadership team. We don’t let these things slide; every incident is addressed in detail. As a coordinator, there’s no such thing as an unresolved violent event. It’s handled immediately.

Principals also established procedures to protect their staff. Nadav (school principal) required that teachers never meet parents alone, ensuring shared responsibility and support: “I insist that teachers hold conversations with parents in pairs, never alone. Even if the meeting seems harmless, it’s better to have two people present, so the teacher doesn’t feel isolated.”

When teachers confronted instances of aggression during the school day, it was crucial to maintain a unified front. Sarit (teacher) stressed the emotional impact of collective support in difficult moments:

There will always be challenging students and parents, but what matters is knowing the school is united and has your back. That you’re not the scapegoat. The students and parents come and go, but the team stays, and that support makes all the difference.

#### 3.7.3. Establishing a Positive Social Climate: Enhancing Positive Parental Involvement, Creating a Shared Discourse, and Building Trust

Interviewees also suggested establishing a positive social climate as means of addressing parental violence. In particular, they suggested building trust and fostering collaboration with parents. They indicated trust, open communication, and involving parents in collaborative dialogue as effective ways to support the child’s best interests. Hagit (teacher) explained her approach to viewing parents as partners:

My relationship with parents has generally been good. I’ve been teaching long enough to know it’s no secret that parents are gradually losing authority over their children. When I contact a parent, it’s never to complain. I approach them as a partner so we can work together to help the child.

Building trust required ongoing effort and investment. Tamar (director at counseling psychological services) emphasized the importance of maintaining this relationship: “Parents are a challenging reality. It’s about continuously building and maintaining relationships. The more actively involved parents are in the education system, and the more the school manages that partnership intentionally, the fewer problems you’ll face.”

Nadav (school principal) detailed his practice of inviting parents to engage in the resolution process and mediating disputes among them:

I strongly believe in dialogue, especially in education. It sets an example for resolving disagreements. We invest a lot of time in building trust with parents, inviting them to speak, listening to them, and being flexible. Allowing different ways to solve problems helps build mutual trust.

#### 3.7.4. Conflict Management Training for Teachers

The findings highlight conflict management training as a key strategy in helping teachers handle aggression and prevent escalation. These trainings, often aimed at newer teachers, equipped staff members with communication tools and emotional support to navigate difficult encounters with students and parents, fostering a sense of belonging and safety. Mona (school counselor) illustrated how new teachers receive support and participate in joint workshops with the staff:

We provide mentoring for new teachers, sometimes even entering classrooms to run workshops and hear about their difficulties. These workshops include both new and experienced teachers. The veteran teachers share their experience. … This also helps the new teacher feel accepted, supported, and included.

Ravit (math teacher) described a designated staff member who supports new teachers through regular conversations: “At our school, there’s a teacher whose job is to meet weekly with new staff [members]. … They have a debriefing conversation, discuss their difficulties, including challenges with others in the system, and she helps them.”

Some schools had developed practical tools to help teachers manage such situations. Rina (director of counseling psychological services) explained her work: “As part of our training on school climate, I talked about conflict management tools, how to manage conflict, and prevent escalation. Everyone brings their own stress, frustrations, and emotional thresholds, and that crashes with the other side’s limitations.” She emphasized two key dimensions in coping: personal support for the affected teacher and a systemic response to restore trust and stability.

When an incident occurs, the teacher is often deeply hurt, there’s real emotional damage. The system must support her personally, whether through the counselor, psychologist, superintendent, or principal. If she feels seen and backed, recovery is more likely. On a systemic level, we must restore trust and safety to the entire school community. The focus is on creating processes of dialogue, conflict management, and organizational resilience.

Natalie (school principal) emphasized the importance of empathy and communication in preventing conflict:

Sometimes, we need to put ourselves in the parents’ shoes, and that resolves many conflicts. When the teacher understands the other side, it doesn’t escalate into aggression. You need to bring them to your side. We’re not at war; we’re all working for the [sake of the] child.

## 4. Discussion

Although violence directed at teachers has gained increasing research, policy, and public attention during the past decade and a half, very limited research has considered parental violence against teachers. To address this gap, this study used in-depth interviews with 46 participants, including teachers, school principals, and policymakers in Israel, to explore how parents’ violence toward school teachers manifests and in which contexts, associated factors, and strategies that teachers and schools use to manage these encounters.

### 4.1. Common Forms of Parental Violence Toward Teachers

Consistent with prior research (e.g., McMahon et al., 2024), the present findings suggest that parental violence against teachers predominantly manifests through verbal abuse. Interviewees recounted numerous instances in which parents employed abrasive, condescending, and aggressive language, often in response to disciplinary actions taken by teachers due to student misbehavior. These results underscore the potential volatility of disciplinary practices, which can provoke parental interference and escalate to aggressive behavior toward educators. Teachers’ efforts to educate children, establish boundaries regarding appropriate behavior, and address issues related to students’ grades often resulted in violence in the form of threats and stalking—either in person or via electronic platforms, particularly WhatsApp—with the intent to undermine their professional authority.

These findings corroborate previous research indicating that disciplinary issues with students are the primary catalyst for parental violence directed at teachers (Tiesman et al., 2013). They echo prior evidence that parents’ concerns about perceived unfairness or inconsistent enforcement of school discipline practices were identified as the most significant factor contributing to conflicts with teachers, which can escalate to violence against them (May et al., 2010).

The current findings underscore threats as a significant concern that teachers face from parents. Such threats not only undermined the professional effectiveness of these teachers but also hindered their ability to perform their duties, discouraging them from addressing student misconduct. Parents often threatened teachers with escalation to the school principal, Ministry of Education, or even the media to exert influence or intimidate. Such threats circumvented teachers’ authority, further eroding trust and intensifying tensions in the already fragile relationships between educators and parents, ultimately hindering effective parent–teacher communication.

Consistent with prior research (Badenes-Ribera et al., 2022), physical violence by parents was the least reported form of aggression among these teachers. Although physical violence is typically considered more severe than verbal abuse or threats, teachers tend to report victimization primarily when it involves physical harm and are less likely to report incidents of nonphysical victimization (Moon et al., 2020). Nonetheless, the current results suggest that verbal abuse and threats also have significant negative consequences for teachers, including emotional distress and an increased sense of insecurity, undermining the teachers’ ability to educate and nurture students effectively.

This research enhances our understanding of how parental violence against teachers manifests, emphasizing that teachers frequently identified parental interruptions as a common form of disruptive and aggressive behavior. This included parents barging into classrooms or interrupting lessons, which is particularly troubling, disruptive, and humiliating for educators. Consistent with prior evidence (Badenes-Ribera et al., 2022), such intrusive acts of violence by parents represent particularly severe forms of teacher-directed aggression that require targeted prevention and intervention efforts.

### 4.2. Multiple Factors Contributing to Parental Violence Against Teachers in the School Ecology

Consistent with prior research (Astor & Benbenishty, 2019; McMahon et al., 2017), the current findings indicate factors across multiple levels in the school ecology that are associated with teachers’ victimization. At the individual level, teachers’ personal characteristics may affect their vulnerability to parental aggression. Specifically, resembling prior research (Martinez et al., 2016; McMahon et al., 2014), teachers with more experience and seniority were found to be less susceptible to parental hostility. It is possible that parents hold greater respect for veteran teachers with extensive teaching experience. It is also likely that experienced teachers project a sense of confidence during interactions with parents, especially in challenging or potentially volatile situations. The findings also suggest that teachers who can establish boundaries with parents experience less violence from them. It appears that just as teachers need to set clear boundaries with students to avoid victimization by students (Berkowitz et al., 2022; McMahon et al., 2020), they should also employ explicit boundary-setting strategies when dealing with parents.

Beyond teachers’ individual traits, the findings emphasize the role of the school as an organization in influencing the risk of parental violence. Teachers noted that although practical tools exist for managing student violence (e.g., sanctions against students), they have no comparable means to address parental aggression. Teachers cannot punish parents, nor can they suspend students for their parents’ violence, leaving teachers vulnerable and exposed without effective strategies to counteract parental misconduct. Thus, beyond sanctions, alternative strategies are necessary for effectively addressing parental aggression toward teachers.

Similar to prior research (e.g., Berkowitz et al., 2022; McMahon et al., 2020), the school’s organizational climate has also been highlighted as important in predicting parental violence toward teachers. The findings underscore the critical importance of collegial, managerial, and administrative support in preventing and managing parental violence. Teachers emphasized that strong backing from school leaders is essential when parents become aggressive, and a lack of support can intensify the violence teachers face. Fostering a positive organizational climate—aligned with the school’s core values, vision, and philosophy—is vital for improving safety and lowering violence (Astor & Benbenishty, 2019). Research on teachers’ victimization has indicated that fostering a positive organizational climate should be an ongoing and integral process, rather than a sporadic effort. Consistently reinforcing a supportive collegial environment helps build trust, promote collaborative relationships, and establish clear norms for behavior, all of which are crucial for effectively preventing and addressing violence against teachers (Berkowitz et al., 2022; McMahon et al., 2020).

The organizational climate of a school transcends the school setting and extends to the policy level (Astor & Benbenishty, 2019). Interviewees criticized the Ministry of Education for providing insufficient support, which hampered efforts to protect teachers effectively. The current findings reveal that in an organizational climate characterized by a culture of ignoring and not addressing problematic issues, coupled with a lack of willingness or effective tools to confront parents and provide support from the school level to the Ministry of Education, the risk of parental violence toward teachers is markedly amplified.

The research findings further underscore the significance of the community as a factor linked to parental violence toward teachers. Specifically, the results reveal a complex pattern of parental attitudes and behaviors. On one hand, parents tend to exhibit a hovering, overprotective style characterized by intrusive involvement in teachers’ educational and instructional work, meddling in their responsibilities and causing harm. On the other hand, interviewees highlighted that contemporary parenting is marked by a notable decline in parental authority, a blurring of intergenerational boundaries, and efforts to shift educational responsibilities onto schools and teachers. This shift has diminished parents’ sense of accountability for their children’s upbringing and intensified conflicts between parents and teachers, ultimately exacerbating parental aggression toward educators. These results are in line with prior research evidence suggesting that reduced parental authority over children is linked to a rise in teacher-directed violence (Berkowitz et al., 2022; McMahon et al., 2020). Similarly, excessive parental involvement and interference in teachers’ educational work may also elevate the likelihood of parental violence against educators (Tiesman et al., 2014).

The current findings further underscore the influence of community socioeconomic status on parental aggression toward teachers. Specifically, the results suggest that in communities with higher socioeconomic status, parents are more likely to employ intimidation tactics, often including legal threats that undermine teachers’ professional authority and decision-making. These findings align with prior research indicating that, particularly in more affluent communities, teachers perceive parents as sources of threat, often manifesting as verbal aggression, attacks, and dismissive or critical behaviors that can undermine teachers’ career advancement and challenge their professional judgment (Addi-Raccah & Grinshtain, 2018). Consistent with the current findings, previous research has documented instances in which parents threatened teachers’ employment by approaching the school board or the teacher’s supervisor or threatening to initiate legal action (McMahon et al., 2023).

Finally, the interviewees in this study observed that similar to broader societal trends, some parents show a lack of respect for the teaching profession and teachers personally. They highlighted that the prevailing perception among both parents and students is that anyone can become a teacher, and this attitude reflects a dismissive view that diminishes the perceived value of educators. Consequently, such attitudes contribute to a heightened risk of parental violence against teachers, because this gap in respect often facilitates aggressive behaviors. These findings match prior evidence suggesting that diminished respect for and undervaluation of the teaching profession in society can cultivate adverse attitudes, hostility, and aggressive behaviors toward educators (McMahon et al., 2020).

#### Positive School Climates and Mentoring for Conflict Management Are Effective Coping Strategies

The results reveal coping mechanisms and strategies that may reduce parental violence toward teachers. Overall, these suggested approaches underscore the importance of fostering a positive social and organizational school climate as an effective means of addressing the issue. Similar to prior research indicating that suspensions are generally ineffective in reducing school violence (Skiba et al., 2022), interviewees noted that suspension is impractical in teacher–parent conflicts. Instead, cultivating a positive school environment appears to yield more constructive outcomes. Consistent with prior research (Martinez et al., 2016; Payne et al., 2003), the findings suggest that cultivating a positive organizational climate—characterized by a unified stance among teachers and staff members, mutual support and solidarity, and the implementation of clear boundaries and behavioral expectations for both students and parents—offers a promising strategy for reducing parental violence toward teachers.

Elements of a positive organizational climate, such as collegial support and backing, enhance teachers’ social capital—defined as their network of institutionalized social connections, relationships, and resources (Adler & Kwon, 2002). Thus, a supportive organizational environment may provide teachers with the additional resources and backing necessary to protect themselves from demanding and threatening parents, as they benefit from strengthened support and legitimacy from colleagues and school leaders. A key strategy identified in the study was that in any meeting with parents—regardless of how benign it may seem—an additional teacher would join, ensuring that no teacher faced parents alone. Conversely, a toxic organizational climate can not only intensify parental aggression due to inadequate response and poor management but also contribute to further victimization, because teachers endure additional harm and humiliation when colleagues and school leadership diminish or dismiss their experiences of victimization.

In line with McMahon et al.’ (2017) findings, the current study underscores the pivotal role of the school principal in shaping a supportive organizational climate. The school leader establishes the tone for the entire school community, has the capacity to mobilize the staff toward fostering a culture of mutual support among colleagues (Astor et al., 2009), and thus possesses the greatest authority to mitigate incidents of parental violence against teachers. The school’s social climate is also an important tool to tackle teachers’ victimization. A prominent aspect of the school’s social climate highlighted in this study is parental involvement, collaboration, and sustained positive communication with parents.

The findings indicate that engaging parents, through efforts to build trust, invite their participation, and integrate them into the school’s coalition, requires an ongoing and sustained process. This continuous engagement serves as a vital strategy in mitigating parental violence, emphasizing that such involvement should not be sporadic or reactive but rather a consistent component of the school’s relational framework. A key aspect of positive collaboration with parents is recognizing that parental violence toward teachers often originates from underlying frustration, typically arising from disputes over disciplinary practices and pedagogical decisions, which can serve as significant sources of tension between parents and teachers. Interviewees emphasized that school staff members should respond with empathy to parents’ concerns, because this approach is essential for sustaining a productive and constructive partnership. Epstein (2019) suggested that effective collaboration fosters an environment in which curiosity and dialogue thrive, allowing for open discussions and healthy debates. They are supported by established frameworks and procedures that facilitate conflict resolution and ensure the partnership’s continuity. Importantly, such partnerships often become even more resilient and robust following periods of disagreement or conflict (Epstein, 2019). Additionally, school leaders must develop, implement, and rigorously enforce comprehensive partnership policies, because teachers require access to information and resources to establish and sustain effective and productive collaborations, even in the presence of conflict (Lasater, 2016).

Finally, the results suggest that to manage conflicts with parents effectively, particularly for newer teachers, mentoring, guidance, and ongoing support are crucial. Such interventions have been shown to reduce incidents of school violence, including violence directed at teachers (Berkowitz et al., 2022). New teachers often face significant challenges in isolation, having to navigate successes and setbacks without sufficient support (Ingersoll & Strong, 2011). Mentoring can enhance their ability to manage conflicts with parents, preventing escalation and thus reducing the risk of parental violence. Schools that provide structured, systematic support tend to offer more consistent assistance tailored to the specific challenges that teachers encounter. This organizational approach aims to improve overall school functioning and foster healthier parent–teacher interactions, ultimately reducing parental aggression. Providing these resources to novice and less experienced educators can strengthen their conflict management skills and contribute to a safer, more inclusive school environment.

### 4.3. Study Limitations and Future Research Directions

Although this study provided valuable insights, several limitations should be acknowledged. The research relied exclusively on the perspectives of educators and policy-level managers regarding parental violence toward teachers, and future studies should incorporate viewpoints from parents and students to gain a more comprehensive understanding, because they may offer alternative explanations. Employing a mixed-methods design could garner deeper insights regarding this relatively underexplored phenomenon. Another limitation of this study is the relatively homogenous sample, which primarily included Hebrew- and Arabic-speaking Israeli educators and policy-level managers. Repeating this research in other regions and contexts could provide a broader understanding of parental violence toward teachers and enhance the generalizability of the findings. Future studies involving diverse populations across different cultural and linguistic backgrounds would contribute to a more comprehensive understanding of this phenomenon globally. Additionally, the sample primarily consisted of nonminority educators and policy makers. Because the nature and severity of violence against teachers can vary across ethnic and cultural contexts, it is recommended that future research include a more diverse and representative sample to examine ethnocultural influences on parents’ violence toward teachers.

### 4.4. Practice and Policy Implications

Our findings indicate that parental violence against teachers is a multifaceted issue rooted in various dynamics in the school environment and broader societal context. Therefore, addressing this problem requires interventions across multiple levels—individual, school, community, and society—because focusing solely on personal or school-based factors without considering prevailing community attitudes, values, and beliefs is unlikely to yield meaningful change. This research highlighted that parental aggression often reflects a broader decline in societal respect for the teaching profession. Effective solutions may involve efforts to enhance the status of educators, such as improving compensation and promoting cultural norms that honor and support teaching. Ultimately, resolving this complex challenge cannot be the sole responsibility of educators or schools. Instead, it demands a community-wide and societal approach—fostering a cultural shift that prioritizes school safety and recognizes the influence of societal norms and values that shape attitudes toward education and authority.

A more positive school climate is likely to enhance overall safety for teachers. It is essential for students, parents, and educators to establish and clearly communicate behavioral expectations and acceptable conduct collaboratively. Implementing transparent and equitable policies against all forms of violence, along with consistent enforcement, is crucial (Berkowitz et al., 2017). Although the importance of school climate is increasingly acknowledged in educational reform efforts, greater focus should be given to fostering the school’s organizational structure, social cohesion, and emotional environment. Additionally, providing mentoring, guidance, and ongoing support to novice teachers is vital, because the findings indicate they are more vulnerable to experiencing parental violence.

## Figures and Tables

**Table 1:** Participants’ Demographic and Professional Characteristics (N = 46).

	Sex	Position	Education	Years of Experience	School	Hebrew or Arabic
1	F	Homeroom and biology teacher	MA	27	High school	Hebrew
2	F	Homeroom teacher	MA	21	High school	Hebrew
3	M	History teacher	MA	7	High school	Hebrew
4	M	Physics teacher	MA	4	High school	Hebrew
5	F	Homeroom teacher and class coordinator	BA	20	High school	Hebrew
6	F	Civics teacher	BA	30	High school	Hebrew
7	F	Homeroom and English teacher	BA	10	High school	Hebrew
8	F	Homeroom and special education teacher	BA	10	High school	Hebrew
9	F	Teacher	BA	1	High school	Hebrew
10	M	Physical education teacher	N/A	12	High school	Hebrew
11	F	Homeroom teacher and counselor	MA	8	High school	Hebrew
12	F	Homeroom and literature teacher	N/A	18	High school	Hebrew
13	M	Physical education teacher	Teaching certificate	6	High school	Hebrew
14	M	Biology teacher	PhD	5	High school	Hebrew
15	F	Teacher and vice principal	N/A	15	High school	Hebrew
16	F	Teacher	Teaching certificate	6	High school	Hebrew
17	F	Mathematics teacher	MA	3	High school	Hebrew
18	F	Homeroom and special education teacher	MA	30	High school	Hebrew
19	F	Homeroom and language arts teacher	N/A	3	High school	Hebrew
20	F	History and civics teacher	N/A	15	High school	Hebrew
21	F	Teacher and class coordinator	N/A	18	High school	Hebrew
22	F	Homeroom and mathematics teacher	MA	16	High school	Hebrew
23	F	English teacher	MA	4	High school	Hebrew
24	F	Language arts teacher	BA	7	High school	Hebrew
25	F	English teacher	BA	40	High school	Hebrew
26	F	Special education teacher	MA	30	High school	Hebrew
27	M	English teacher	MA	3	High school	Hebrew
28	F	Homeroom and Hebrew teacher	MA	25	High school	Hebrew
29	F	Homeroom and biology teacher	N/A	16	High school	Hebrew
30	F	Teacher and counselor	MA	22	High school	Hebrew
31	F	Electronics teacher and counselor	MA	17	High school	Arabic
32	F	Arabic language and homeroom teacher	BA	6	High school	Arabic
33	F	English teacher	MA	6	Middle school	Hebrew
34	F	Homeroom teacher	N/A	4	Middle school	Hebrew
35	F	English teacher	PhD	23	Middle school	Hebrew
36	F	Mathematics teacher	MA	14	High school	Arabic
37	F	School principal	N/A	30	Middle school	Hebrew
38	M	School principal	High school	20	High school	Arabic
39	M	School principal	PhD	15	High school	Hebrew
40	F	School principal	MA	17	High school	Hebrew
41	F	Director	PhD	12	Counseling psychological services	N/A
42	F	Director of programs and prevention division	MA	9	Counseling psychological services	N/A
43	F	Director of unit for promoting positive school climate and reducing violence	MA	7	Counseling psychological services	N/A
44	M	Chair	MA	22	Teachers union	N/A
45	F	Pedagogical coordinator	PhD	7	Teachers union	N/A
46	M	Pedagogical coordinator	MA	8	Teachers union	N/A

## Data Availability

The data presented in this study are available on request from the first author to ensure the privacy of the participating schools and teachers.
